# Socioeconomic Status and Fine‐Grained Literacy Outcomes in Heritage Bilingual and Monolingual Children With and Without Specific Learning Disorders

**DOI:** 10.1002/dys.70051

**Published:** 2026-10-09

**Authors:** Paola Bonifacci, Elisabetta Lombardo, Marina Porrelli, Alessia Rapino, Anna Gallani, Carlotta Facini, Chiara Gelmini, Chiara Nanni, Michela Muccinelli, Elena Mannino, Simona Chiodo

**Affiliations:** ^1^ Department of Psychology University of Bologna Bologna Italy; ^2^ UO NPIA, AUSL Bologna Bologna Italy; ^3^ Polo Provinciale DSA AUSL Modena Modena Italy; ^4^ Centro DSA AUSL Ferrara Ferrara Italy; ^5^ UO NPIA, AUSL Parma Parma Italy; ^6^ NPIA, AUSL‐IRCCS Reggio Emilia Reggio Emilia Italy; ^7^ UO NPIA, AUSL Imola Imola Italy; ^8^ AUSL Romagna Forlì Italy

**Keywords:** cognitive profile, heritage bilingualism, language history, socioeconomic status, specific learning disorders

## Abstract

This study investigated the sociolinguistic background and the cognitive and literacy profiles of heritage bilingual (HB) children with specific learning disorders (SLD) in reading and spelling or typical development (TD) in comparison with their monolingual peers. About 454 primary school children were included and divided into four groups: HBs with SLD (*n* = 108) or TD (*n* = 70), and monolinguals with SLD (*n* = 106) or TD (*n* = 170). Children completed tasks assessing nonverbal IQ, working memory, processing speed, nonword repetition, listening comprehension, and literacy skills, including word, nonword, and text reading and spelling, and reading comprehension. Parents provided information on language history and socioeconomic status (SES). Results showed comparable sociolinguistic profiles across groups, with greater heritage language maintenance in the HB‐TD group. SES primarily predicted performance on tasks with higher lexical demands and nonverbal IQ. Once SES was considered, language background only influenced spelling tasks. Interaction effects indicated a stronger influence of SES in HBs. At the phenotypic level, HBs and monolingual children with SLD showed comparable profiles, with an SLD‐specific disadvantage for HBs emerging only in nonword spelling. These findings underscore the importance of SES in assessing HB children and promoting the home language in clinical populations.

## Practitioner Points

- Heritage bilingual children may be at increased risk of heritage‐language erosion.
- Tasks with high lexical demands are particularly sensitive to SES differences in bilingual learners.
- When SES is considered, bilingualism alone does not account for most cognitive and literacy differences.
- Heritage bilingual children with SLD generally show profiles comparable to monolingual peers, except in nonword spelling.
- Spelling skills may require more time to consolidate in bilingual children.

Many children learn to read and write in a language other than their home language, often a minority language with limited social recognition or classroom use. Known as heritage bilinguals (HBs) (Paradis 2023), these students often come from immigrant backgrounds and vary widely in language exposure and socioeconomic status (SES). Some HBs, independently of language background and similarly to their monolingual peers, may present specific learning disorders (SLDs), which call for accurate identification and tailored support. SLDs are neurodevelopmental conditions affecting academic skills such as reading, spelling, or mathematics, arising from multiple interacting factors (McGrath et al. 2020), and are best understood within a multilevel perspective (Zoccolotti 2020). Diagnosis is based on standardised testing and a comprehensive review of medical and educational history, ruling out intellectual disability, sensory impairments, and inadequate instruction (APA 2013). Catts et al. (2024) highlight challenges in redefining dyslexia, including risks of misidentifying children from minority backgrounds, which may affect access to appropriate support (Terry et al. 2023).

Identifying SLDs in HB children is complicated by several potential biases: (1) literacy acquisition in a second language often follows a different timeline than in monolinguals, depending on task demands; (2) individual language history factors, such as age of first exposure and language use (Paradis 2023); and (3) the confounding impact of SES, which significantly influences literacy outcomes (Bonifacci et al. 2022). This study compares HBs and monolingual children, both with and without SLDs, a population still relatively underexplored in bilingual literacy research, to examine differences in their literacy and cognitive profiles while accounting for language history and SES.

## 1 Literacy Trajectories in Bilinguals and the Identification of SLD

One consistent finding is that, within 2 years of schooling, bilinguals and monolinguals perform similarly on nonword decoding tasks (August and Shanahan 2006; Bellocchi et al. 2017; Bonifacci and Tobia 2016; Droop and Verhoeven 2003), indicating strong sublexical reading skills in bilinguals. This aligns with evidence that nonword repetition, a key diagnostic tool for Developmental Language Disorder (DLD) (Ortiz 2021), is typically mastered by bilinguals within 2 years of exposure. In terms of word reading accuracy, HBs perform comparably to monolinguals in both opaque (e.g., English) and transparent orthographies (e.g., Dutch and Italian) (August and Shanahan 2006; Droop and Verhoeven 2003; Bellocchi et al. 2017; Bonifacci and Tobia 2016). However, they may lag behind in reading low‐frequency or orthographically complex words (Droop and Verhoeven 2003; Bellocchi et al. 2016). This gap may reflect greater reliance on the sublexical (phonological) reading route among bilinguals, whereas monolinguals may rely more efficiently on the lexical reading route (Coltheart et al. 2001), consistent with their richer vocabulary knowledge in the language of schooling (Bialystok 2009) and the higher quality of lexical representations supporting efficient word recognition (Perfetti 2007).

For spelling, a meta‐analysis by Zhao et al. (2016) found that bilinguals tend to outperform monolinguals in real‐word spelling. However, studies conducted in transparent orthographies such as Dutch, German, and Italian show that HBs generally perform worse than monolinguals in word spelling through the end of primary school (Affranti et al. 2024; Verhoeven 2000; Zhang et al. 2022), even after accounting for SES (Bonifacci et al. 2022; Artuso et al. 2024). No such difference was observed in children with developmental language disorders (de Bree et al. 2025). Interestingly, this spelling gap was not present in multilingual children schooled in Italian when performing English spelling tasks, where their exposure was equivalent to that of monolingual peers (Bonifacci et al. 2017).

In terms of reading comprehension, research suggests that bilinguals may lag behind their monolingual peers (Melby‐Lervåg and Lervåg 2014). However, studies also show that they can achieve comparable levels in both opaque (e.g., Lesaux et al. 2007) and transparent orthographies (Bonifacci and Tobia 2016; Bonifacci et al. 2017). According to the Simple View of Reading (SVR) (Gough and Tunmer 1986; Hoover and Gough 1990), reading comprehension results from decoding and oral language skills. Evidence suggests that bilinguals' challenges are more closely related to oral language, particularly vocabulary, than to decoding skills (Melby‐Lervåg and Lervåg 2014; Bonifacci and Tobia 2017).

Finally, the existence of a bilingual advantage in cognitive processing remains controversial (e.g., Lowe et al. 2021), with limited evidence from traditional cognitive assessments. Some studies report bilingual advantages in nonverbal tasks such as block design (Bialystok and Majumder 1998; Lauchlan et al. 2012), while others find no significant differences (Karlsson et al. 2015) or effects of SES on cognitive outcomes in bilingual and monolingual children with neurodevelopmental disorders (Peristeri et al. 2022). A previous study (Riva et al. 2016) directly compared HBs and monolingual peers with SLD and found similar performance in word, nonword, and text reading speed, but lower text reading accuracy in HBs. In spelling, bilinguals lagged in word spelling, but not in nonword spelling. On the WISC‐IV, HBs scored lower on the working memory index but performed comparably on verbal comprehension, perceptual reasoning, and processing speed.

Overall, literacy and cognitive development in children with SLDs vary according to language demands, but more research is needed to understand these patterns specifically in multilingual populations.

## 2 Socioeconomic Status and Linguistic History in Relation to Literacy Acquisition

SES is an important factor in children's cognitive, language, and literacy development. A meta‐analysis by Mooney et al. (2021) highlighted a moderate relationship between SES and cognitive outcomes (i.e., working memory), while SES disadvantage has long been associated with lower academic achievement (Bradley and Corwyn 2002). Research shows that SES and bilingualism have distinct effects on children's development. High‐SES children outperform low‐SES peers across a wide range of abilities, including nonverbal tasks (Calvo and Bialystok 2014), highlighting the broad influence of SES. In contrast, bilingualism shows more domain‐specific effects, typically lower vocabulary in the societal language but stronger executive functions. Low‐SES HBs often enter school with weaker L2 skills (Hoff 2013), and bilinguals tend to have smaller L2 vocabularies and weaker verbal short‐term memory, with SES further affecting performance even on tasks that rely less on linguistic knowledge (Meir and Armon‐Lotem 2017). SES also plays a pivotal role in literacy development. Kieffer (2010) found that both English language learners and students from low‐SES backgrounds are at higher risk for late‐emerging literacy difficulties, with similar risks for bilingual and monolingual students of comparable SES. Hassunah‐Arafat et al. (2021) also highlighted SES as a major factor in early literacy, alongside the richness of the Home Literacy Environment (HLE). Schöfl et al. (2024) showed that SES influences spelling skills indirectly through language abilities.

Despite this, few studies clearly distinguish between the effects of SES and bilingualism. Bonifacci et al. (2022) found SES to be more closely linked to reading comprehension at the end of primary school, while bilingualism more strongly affected spelling throughout. Linguistic history also matters: Bonifacci and Tobia (2016) found no differences in reading comprehension between early bilinguals and monolinguals, but late bilinguals performed worse. While both bilingual groups showed similar nonword reading skills, late bilinguals read real words more slowly. Regarding language exposure, Carioti et al. (2024) reported that frequent minority language use correlated with lower accuracy in Italian reading. However, findings on the role of L2 use at home remain mixed, especially for oral skills, where both input quality and richness are relevant (Paradis 2023). However, less research has examined how language history affects literacy in HBs.

## 3 Present Study

This study is part of the broader ADLAB (Assessment of Language and Learning Disorders in Bilinguals) multicenter project, led by LADA Lab (Laboratory for the Assessment of Learning Disorders), University of Bologna, in collaboration with eight public health centres in Northern Italy. This study is grounded in the assumption that SLD (i.e., Dyslexia, spelling disorders), SES, and language background are independent but interacting dimensions. After carefully examining the sociodemographic characteristics of the groups, an aspect often overlooked in the literature, the present study aims to investigate how diagnostic status (SLD vs. typical development), language background (HBs vs. monolinguals), and socioeconomic status (SES) uniquely and jointly shape children's literacy and cognitive linguistic profiles. Across many contexts, socioeconomic disparities disproportionately affect families of migrant background, a pattern that is well documented in Italy (ISTAT 2024). By integrating sociolinguistic, cognitive, and literacy measures within a single analytical framework, this study addresses a critical gap in the literature by enabling a fine‐grained examination of the independent and interactive effects of diagnosis, language background, and SES on reading and spelling skills, and by offering a novel characterisation of literacy profiles in HBs with SLD. Accordingly, the study addresses two main research questions.

Research Question 1 Sociolinguistic background of the study population.

The first aim was to describe the sociolinguistic background of the sample by examining SES, early language milestones, and preschool exposure across monolingual and HB children with and without SLD. Within the bilingual subgroup, we also explored language history (e.g., age of first exposure, use of Italian and the heritage language—HL, and language dominance) to assess whether bilingual experiences varied by diagnostic status. HBs were expected to have lower SES than monolinguals, reflecting well‐documented socioeconomic differences in immigrant families, whereas no SES differences were expected between SLD and TD groups (Hypothesis 1). Although no specific predictions were made regarding language history, we examined whether patterns of exposure and dominance might differentiate HB children with and without SLD, an area where evidence remains limited.

Research Question 2 Multigroup comparison and the role of SES.

The second aim was to examine how SES, language background, and diagnostic status jointly predict literacy and cognitive outcomes. Diagnostic status was expected to show a broad influence on literacy and cognitive measures, particularly in decoding and spelling, consistent with phonological and orthographic profiles associated with SLD (Hypothesis 2a). SES was expected to be positively associated with literacy and language skills, particularly those reflecting environmental and educational input rather than basic decoding (Hypothesis 2b). Bilingualism was expected to show domain‐specific associations, with potential differences in tasks requiring greater lexical or orthographic consolidation (e.g., spelling, text reading), but not a generalised pattern across all literacy outcomes (Hypothesis 2c). Finally, analyses of interactions among SES, bilingualism, and diagnostic status were conducted to determine whether these dimensions exert non‐independent effects on literacy outcomes. We anticipated that interaction terms would yield additional explanatory value beyond the main effects, clarifying whether the association between bilingualism and SLD varies as a function of socioeconomic conditions (Hypothesis 2d).

## 4 Method

### 4.1 Participants

The final sample of the present study consists of 454 children aged 8 to 12 years (mean age = 113.32 months; SD = 10.8; 3rd to 5th grade; 52% female). Of these, 70 were HB children with typical development (HB‐TD; mean age = 113.1 months; SD = 11.01, 52.9% female), 108 were HB children with SLD (HB‐SLD; mean age = 113.82 months; SD = 10.27; 47.2% females), 170 were typically developing monolingual children (Mon‐TD; mean age = 112.62 months; SD = 10.06; 54.1% females), and 106 were monolingual children with SLD (Mon‐SLD; mean age = 114.1 months; SD = 12.33; 52.8% females). Children in the four groups did not differ in age [*F*_(3,450)_ = 0.507, *p* = 0.68] or gender distribution (*χ* ^2^(3) = 1.34, *p* = 0.72). The common inclusion criterion was a nonverbal intelligence quotient (NVIQ) greater than 70 and the absence of sensory and neurological impairments. Additional inclusion criteria were defined for each group.

Heritage bilinguals: Children were considered HB if their parents reported speaking at least one language other than Italian in the family context. There was variability in the languages spoken at home: Arabic 21.3%, Albanian 13.5%, Romanian 12.4%, Urdu 6.2%, Spanish 6.2%, Punjabi 5.6%, Chinese 5.6%, Pidgin English 4.5%, Hindi 3.9%, Russian 3.4%, Tagalog 2.8%, Bengali 2.2%, Other 12.4% (less than 2% each: Wolof, Polish, Twi, Turkish, Tamil, etc.). 87.1% of the parents were both born abroad, 11.2% of the couples had one parent born abroad and only 1.7% had both parents born in Italy. Regarding children, 82.0% were born in Italy, with a similar percentage for the SLD and TD groups (82.4% and 81.4% respectively). More information on language exposure and language use for the HB group is presented in Table 2. For the monolingual group, both parents reported speaking only Italian in the family context.

SLD. Children with SLD were recruited and assessed in seven of the eight public health centres (developmental neuropsychiatric units) participating in the ADLAB research project. Only children diagnosed with reading disorder (dyslexia) and/or spelling disorder (dysorthographia) were included; children with only dyscalculia were excluded, although comorbid math disorder was present in 9.3% of cases. Diagnoses were made according to national guidelines (ISS 2021; PARCC 2011) for SLD, requiring at least two parameters below −2 SD in speed and/or accuracy on word, nonword, and passage reading tests for reading disorder (dyslexia, F81.0, ICD‐10), and at least two parameters below −2 SD on word, nonword, or sentence/passage dictation tests for spelling disorder (dysorthographia, F81.1, ICD‐10).

Children with TD were recruited from schools in northern Italy, with the absence of a formal SLD diagnosis as the inclusion criterion. The initial sample included 489 participants. To reduce assessment bias, the sample was reviewed to exclude false positives and false negatives. SLD participants scoring below −1.5 SD on national reading or writing norms were retained as true positives, whereas TD participants with more than 50% of literacy scores below −1.5 SD were excluded. Five HB and 12 monolingual children were excluded as false positives, and 12 HB and 6 monolingual children as false negatives.

### 4.2 Materials

#### 4.2.1 Background Measure

Socioeconomic status: Parents completed the Hollingshead (1975) to assess SES. Each parent's education (scored 1–7) and occupation (scored 1–9) were combined using the formula: (*education* × 3) + (*occupation* × 5). The average of both parents' scores was used as the child's SES; for single parents, their individual score was used. SES scores ranged from 8 to 66.

Linguistic history: For this study, researchers conducted a parental interview in Italian or, when needed, with language mediators. The first section gathered the child's background: birth details, age of arrival in Italy (if applicable), languages spoken at home, age of first exposure to each language, first words, preschool attendance, and HL schooling. The second section focused on parents, including birth details, migration history, SES, migration plans (stable vs. variable), and self‐rated proficiency in Italian and the HL. The third section addressed language use: parents rated their child's exposure to Italian and the HL across family members and media on a 4‐point scale. Language exposure dominance was calculated as the difference between HL and Italian exposure (negative = more Italian). Parents also rated the child's best language (1 = HL, 2 = Italian, 3 = both) in comprehension, production, and preference.

#### 4.2.2 Cognitive Profile

Nonverbal IQ. Nonverbal reasoning was assessed using the Matrix subtest of the Kaufman Brief Intelligence Test–2 (K‐BIT2; Kaufman and Kaufman 2004; Bonifacci and Nori 2016). Children selected the picture that best completed a visual matrix. Correct responses were scored as 1, with standard scores derived from normative data. The number of administered items varied according to starting points and discontinuation rules. Split‐half reliability is reported as 0.86.

Nonword Repetition. Phonological short‐term memory was assessed using the nonword repetition subtest of the BVL 4–12 (Marini et al. 2015). Children repeated 15 nonwords constructed according to Italian phonotactic rules. Each correctly repeated item was scored as 1, and performance was converted into *z*‐scores based on normative data. The test–retest reliability reported in the manual is 0.72.

Working Memory. Working memory was measured using the Working Memory Index (WMI) of the WISC‐IV (Wechsler 2003), including the Digit Span and Letter–Number Sequencing subtests. Children repeated sequences of numbers (forward and backward) and reordered mixed letter–number strings. Raw scores were converted to standard scores using test norms.

Processing Speed. Processing speed was assessed with the Processing Speed Index of the WISC‐IV (Wechsler 2003), including Coding and Symbol Search subtests. Both tasks were timed (2 min), and raw scores were converted to standard scores according to the manual.

#### 4.2.3 Literacy Tasks

Decoding. Word, nonword, and passage reading were assessed using the ALCE battery (Bonifacci et al. 2014). Word reading included three lists (20 words each) of increasing length and decreasing frequency, and nonword reading included two lists (15 nonwords each). Reading speed (syllables per second) and accuracy (percentage of errors) were computed. Passage reading involved reading a grade‐level text aloud followed by comprehension questions. *Z*‐scores were derived from test norms. Reported reliability was 0.89 for word reading and 0.96 for nonword reading.

Spelling and Dictation. Word and nonword spelling were assessed using the BVDDE‐2 (Sartori et al. 2007), in which children wrote words and nonwords dictated aloud; errors were scored as incorrect responses, and *z*‐scores were derived from norms (reliability = 0.68). Passage dictation was assessed with the BVSCO‐2 (Tressoldi et al. 2013), using grade‐specific texts; each misspelled word was scored as an error, and performance was converted to *z*‐scores.

Comprehension. Listening and reading comprehension were assessed using the ALCE Battery (Bonifacci et al. 2014) with grade‐level passages followed by 10 open‐ended questions. In listening comprehension, the text was read aloud by the examiner, whereas in reading comprehension the text remained available. Internal consistency ranged from 0.68 to 0.80 for listening comprehension and from 0.74 to 0.83 for reading comprehension.

### 4.3 Procedure

Parents signed an informed consent form to participate in the study. The matrix subtest, spelling and reading comprehension tasks were administered collectively in class (for TD groups) or individually for SLD groups. All other tasks were administered individually by the experimenters. The battery took approximately 2 h to complete. The study was approved by the Ethics Committee for each site of the multicenter study (632‐2021‐OSS‐AUSLBO; 977‐2021‐OSS‐AUSLBO; 900/2021/Oss/AUSLF; 734‐2021‐OSS‐AUSLIM; 840/2021/OSS/AUSLMO; 848/2021/OSS/AUSLPR; 971/2021/OSS/AUSLPC; 8540/2021/I.5/228/Cerom; 869/2021/OSS/AUSLRE).

### 4.4 Data Analysis

Analyses were conducted on age‐adjusted standard or *z*‐scores derived from test manuals. For literacy measures, outliers were handled using Winsorisation (Wilcox 2010). All variables met normality assumptions. Some missing data were present for language history variables; corresponding sample sizes are reported in Table 2. Group differences in demographic and language background variables were examined using ANOVAs with diagnostic group (SLD vs. TD) and language background (HB vs. monolingual) as factors (SPSS 29.0.2.0). Language history variables, available only for HBs, were analysed using Welch *t*‐tests to compare SLD and TD groups (Table 2).

To address the second research question, preliminary ANOVAs including diagnostic group and language background as main effects were conducted (see Supporting Information). Separate General Linear Models (GLMs) were then run in Jamovi (v. 2.7, 2025) for each cognitive and literacy outcome, with diagnostic group and language background as between‐subject factors and child composite SES entered as a covariate. Main effects of diagnostic group, language background, and SES, as well as their two‐way interactions, were examined. Significant interactions were followed up with simple effects analyses.

## 5 Results

### 5.1 Research Question 1. Sociolinguistic Background of the Study Population

Table 1 reports descriptives for socioeconomic status, first words, and preschool attendance in the four groups, whereas Table 2 reports language history values for the HB groups (TD and SLD).

**TABLE 1:** Descriptive statistics (mean, SD and valid cases) for socioeconomic status, first words and preschool attendance in the four groups.

	HB‐TD			M‐TD			HB‐SLD			M‐SLD		
	Mean	SD	*N*	Mean	SD	*N*	Mean	SD	*N*	Mean	SD	*N*
Education mother	3.60	1.55	70.00	4.31	1.35	170.00	2.99	1.54	108.00	3.96	1.33	106.00
Education father	3.34	1.38	68.00	3.87	1.33	170.00	2.85	1.34	107.00	3.40	1.33	104.00
Occupation mother	2.19	1.96	67.00	5.02	2.28	169.00	1.94	1.53	108.00	4.13	2.38	106.00
Occupation father	3.49	1.62	67.00	4.98	1.98	170.00	3.03	1.07	106.00	4.14	1.74	103.00
Child's SES	24.60	10.90	70.00	37.20	11.30	170.00	21.10	7.40	108.00	31.60	11.40	106.00
First words Italian^a^	27.38	20.47	61.00	13.63	6.44	167.00	29.14	25.05	106.00	11.20	4.63	101.00
Preschool attendance^a^	24.99	14.90	68.00	34.65	11.35	156.00	26.12	12.77	104.00	34.84	10.57	105.00

^a^ Months.

**TABLE 2:** Descriptive statistics (mean, SD and valid cases) for linguistic history variables in HBs group.

	TD			SLD			Statistics			
	Mean	SD	*N*	Mean	SD	*N*	*t*	df	*p*	Cohen's *d*
Age of arrival (Italy)^a^	6.96	19.64	70	6.21	17.75	108	0.26	136.72	0.80	0.04
Age of first exposure L2^a^	21.54	26.35	70	25.43	22.37	108	−1.02	130.20	0.31	−0.16
Total length of exposure^a^	91.56	28.31	70	88.40	21.16	108	0.80	118.05	0.43	0.13
First words HL^a^	14.03	8.38	60	14.60	10.03	106	−0.39	141.34	0.70	−0.06
L2 proficiency (mother)	3.59	1.12	70	3.15	1.09	108	2.57	144.53	0.01*	0.40
L2 proficiency (father)	3.94	1.01	64	3.53	1.17	106	2.41	148.73	0.02*	0.38
Exposure HL *minus* L2 (mother)	0.09	1.78	70	0.20	1.90	108	−0.42	154.71	0.67	−0.06
Exposure HL *minus* L2 (father)	0.00	1.96	68	−0.01	2.12	105	0.03	151.15	0.98	0.01
Exposure HL *minus* L2 (siblings)	−1.05	1.60	60	−1.62	1.71	94	2.09	132.01	0.04*	0.34
Exposure HL *minus* L2 (TV‐media)	−1.43	1.44	70	−2.00	1.55	106	2.50	155.18	0.01*	0.38
Father, years in Italy	20.76	8.39	62	21.51	6.72	100	−0.60	108.58	0.55	−0.10
Mother, years in Italy	17.04	8.06	69	16.83	6.27	105	0.19	120.46	0.85	0.03

^a^ Months.

Univariate analysis of SES showed significant main effects for language background [*F*(1, 450) = 124.91, *p* < 0.001, *ηp* ^2^ = 0.22] and diagnostic group [*F*(1, 450) = 19.44, *p* < 0.001, *ηp* ^2^ = 0.04], with no significant interaction [*F*(1, 450) = 0.98, *p* = 0.32, *ηp* ^2^ = 0.002]. Bonferroni post hoc tests revealed that SLD groups had lower SES than TD groups, and HB children had lower SES than monolinguals (see Figure 1).

**FIGURE 1:**
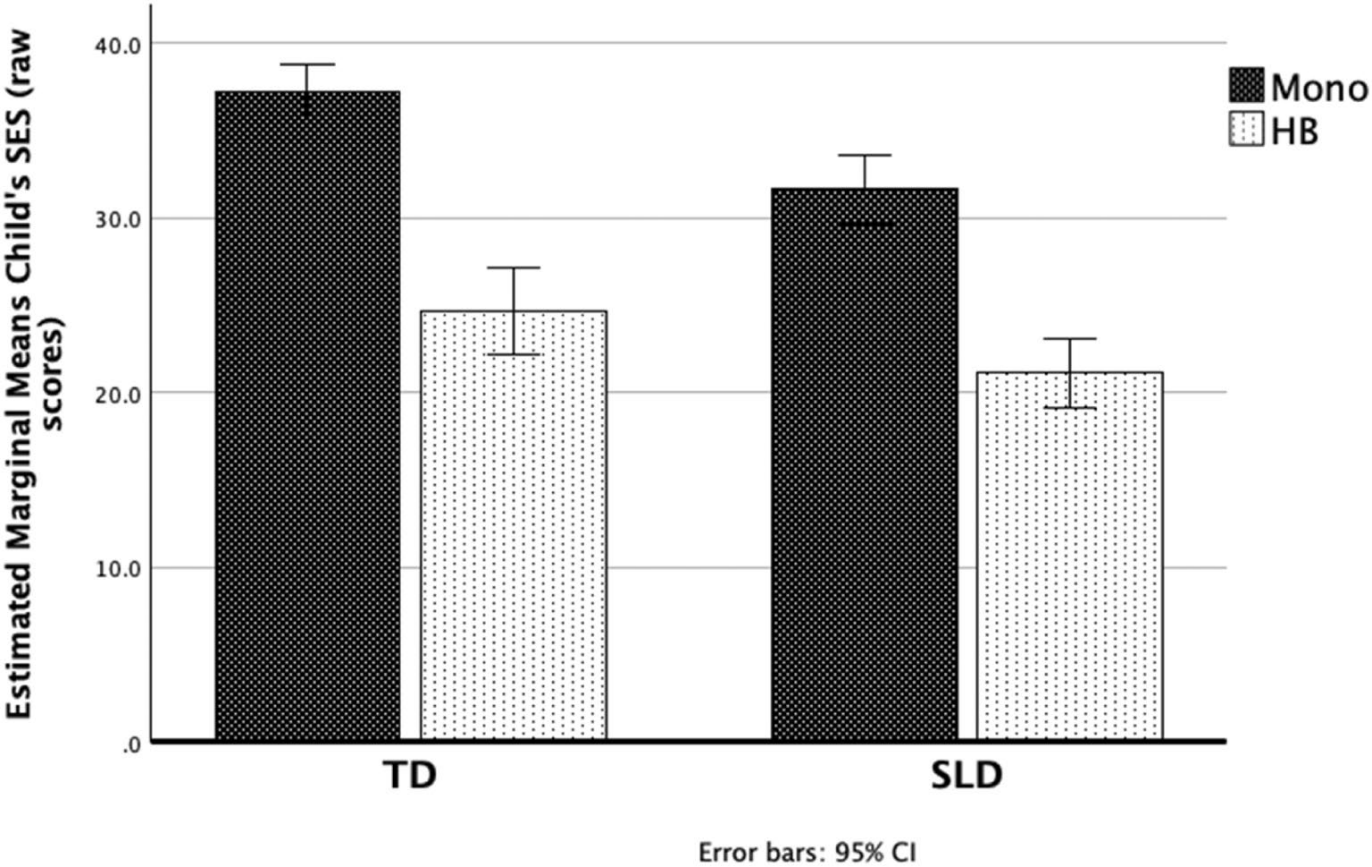
SES differences across HB and monolingual peers with or without SLD.

Similar patterns emerged when analysing individual SES components. A MANOVA revealed main effects of SLD [*F*(4, 435) = 6.04, *p* < 0.001, *ηp* ^2^ = 0.05] and language background [*F*(4, 435) = 37.48, *p* < 0.001, *ηp* ^2^ = 0.26], but no interaction [*F*(4, 435) = 1.70, *p* = 0.15, *ηp* ^2^ = 0.02]. Univariate results showed consistent effects across maternal and paternal education and occupation. Thus, only the composite child SES score was used in further analyses. No significant effect of SLD was found on age of first words [*F*(1, 434) = 0.046, *p* = 0.831, *ηp* ^2^ = 0.00], but a main effect of language background emerged [*F*(1, 434) = 103.71, *p* < 0.001, *ηp* ^2^ = 0.19], with bilinguals producing first words in Italian later than monolinguals. There was no interaction [*F*(1, 434) = 1.81, *p* = 0.18, *ηp* ^2^ = 0.004]. Regarding preschool exposure (total months in nursery and preschool), only a main effect of language background was found [*F*(1, 432) = 56.97, *p* < 0.001, *ηp* ^2^ = 0.12], with HBs attending less. No effects of SLD [*F*(1, 432) = 0.29, *p* = 0.588, *ηp* ^2^ = 0.001] or interaction [*F*(1, 432) = 0.149, *p* = 0.700, *ηp* ^2^ = 0.001] were observed.

As regards language history, HB‐TD children had higher exposure to the HL, particularly with siblings and through TV/media, than HB‐SLD children. Their parents also reported higher proficiency in Italian. However, no group differences emerged in language use with mothers or fathers. There were no significant differences in other language history variables, such as age of first L2 exposure or years since arrival in Italy (for both children and parents). The groups were also similar in migration plans (TD: 2.9%; SLD: 4.7%; *χ* ^2^(1) = 0.382, *p* = 0.54) and HL literacy experiences (TD: 13.4%; SLD: 12.1%; *χ* ^2^(1) = 0.06, *p* = 0.80). Regarding children's language use and proficiency, HB‐SLD children were more often rated by parents as dominant in Italian for comprehension, production, and language preference. In contrast, HB‐TD children showed a higher proportion of balanced bilingualism (comprehension: *χ* ^2^(2) = 20.87, *p* < 0.001; production: *χ* ^2^(2) = 19.18, *p* < 0.001; preferred language: *χ* ^2^(2) = 21.43, *p* < 0.001; see Figure 2).

**FIGURE 2:**
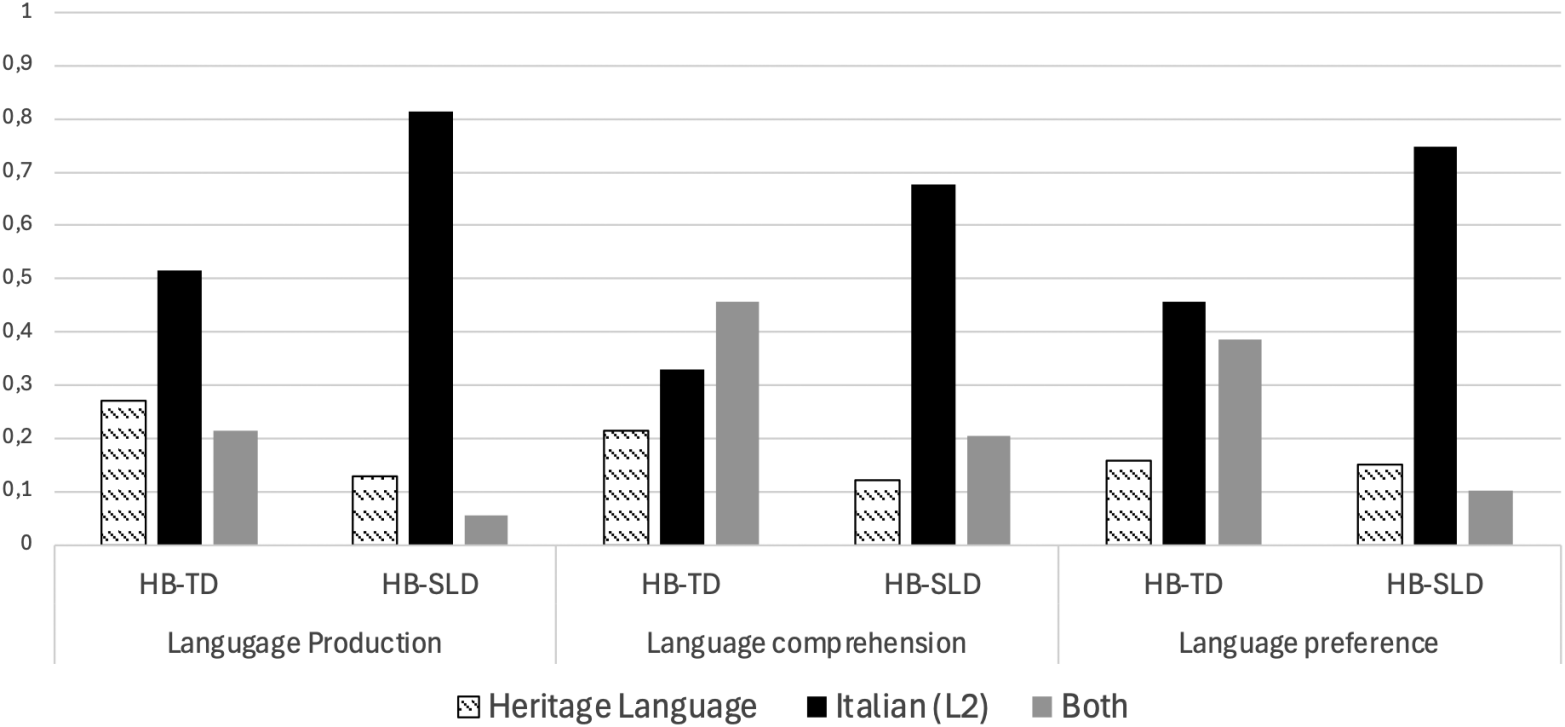
Distribution (%) of language use and preference in HB with TD or SLD.

### 5.2 Research Question 2. Multigroup Comparison and the Role of SES

#### 5.2.1 Preliminary Analysis of Group Comparison Without Controlling for SES

Descriptive statistics for each group for all outcome measures are presented in Table 3.

**TABLE 3:** Descriptive statistics (means, standard deviations, skewness, and kurtosis) for each group for all cognitive and literacy measures.

	Monolingual‐TD		Monolingual‐SLD		HB‐TD		HB‐SLD		Skewness	Kurtosis
	Mean	SD	Mean	SD	Mean	SD	Mean	SD	SE = 0.115	SE = 0.229
Nonverbal IQ*	107.65	14.70	104.23	12.88	104.06	18.51	104.37	9.47	−0.34	−0.05
Working Memory Index*	99.17	13.89	86.64	11.43	93.46	12.24	82.95	11.55	0.41	0.08
Speed of Processing Index*	98.73	15.93	92.21	13.48	97.61	14.85	91.95	17.73	0.02	1.13
Nonword repetition^	0.56	0.39	0.44	0.53	0.50	0.37	0.41	0.53	−1.26	1.93
Listening comprehension^	0.45	0.85	0.04	1.06	0.25	0.79	−0.26	1.01	−0.71	0.01
Word reading speed^	−0.09	0.78	−1.54	0.76	−0.03	0.98	−1.61	0.65	0.283	−0.394
Word reading accuracy^	0.11	0.81	−1.53	2.07	−0.22	0.97	−1.98	2.08	−1.013	0.003
Nonword reading speed^	−0.11	0.78	−1.26	0.73	0.16	0.91	−1.13	0.71	0.368	−0.139
Nonword reading accuracy^	0.21	0.72	−1.38	1.46	0.15	0.74	−1.53	1.67	−0.896	−0.005
Passage reading Speed^	0.00	0.83	−1.77	0.71	0.23	1.14	−1.85	0.76	0.276	−0.449
Passage reading accuracy^	0.11	0.73	−1.50	1.50	−0.28	1.23	−1.78	1.67	−1.1	0.271
Word spelling^	−0.41	1.44	−3.72	2.88	−0.64	1.70	−5.93	3.12	−0.991	−0.08
Nonword spelling^	−0.81	1.26	−0.86	1.16	−0.32	1.00	−1.48	1.48	−0.707	−0.149
Passage dictation^	−0.63	1.42	−4.95	3.04	−1.43	2.28	−7.57	3.39	−0.914	−0.113
Reading comprehension^	0.21	1.03	−0.62	1.17	0.42	1.05	−1.19	1.21	−0.293	−0.423

*Note:* *Standard scores, ^ z‐scores.

Statistical outputs from the preliminary analyses (ANOVAs) are reported in the Supporting Information. No group differences were found in nonverbal IQ. Children with SLD showed broadly weaker cognitive and literacy performance than TD peers, whereas HBs differed from monolinguals mainly in working memory, listening comprehension, and literacy accuracy, while showing comparable or faster performance in reading speed, particularly for nonwords.

#### 5.2.2 General Linear Models (GLMs) With SES and Interactions

Main effects and interactions are reported in Table 4.

**TABLE 4:** General linear models: main effects and interactions.

Outcome	*R* ^2^	*F*	SES (*p*)	Lang (*p*)	Diagn (*p*)	SES × Lang (*p*)	SES × Diag (*p*)	Lang×Diag (*p*)
Nonverbal IQ	0.06	*F*(6,445) = 4.65, *p* < 0.001	**0.001**	0.656	0.633	0.516	**0.014**	0.870
Working Memory Index	0.22	*F*(6,445) = 21.37, *p* < 0.001	**< 0.001**	0.103	**< 0.001**	0.978	0.308	0.693
Processing speed	0.05	*F*(6,445) = 3.60, *p* = 0.002	0.707	0.710	**< 0.001**	0.183	0.342	0.330
Nonword repetition	0.02	*F*(6,421) = 1.18, *p* = 0.315	0.263	0.569	0.111	0.716	0.991	0.352
Listening comprehension	0.10	*F*(6,445) = 7.82, *p* < 0.001	**0.011**	0.111	**< 0.001**	0.608	0.609	0.814
Word reading speed	0.41	*F*(6,445) = 50.89, *p* < 0.001	0.057	0.895	**< 0.001**	0.059	**0.033**	0.679
Word reading accuracy	0.24	*F*(6,445) = 23.79, *p* < 0.001	**< 0.001**	0.623	**< 0.001**	**0.046**	**0.020**	0.082
Nonword reading speed	0.33	*F*(6,445) = 35.88, *p* < 0.001	0.660	0.086	**< 0.001**	0.949	0.651	0.747
Nonword reading accuracy	0.28	*F*(6,445) = 29.34, *p* < 0.001	0.076	0.932	**< 0.001**	0.662	0.732	0.445
Text reading speed	0.46	*F*(6,445) = 62.22, *p* < 0.001	0.223	0.821	**< 0.001**	0.500	0.400	0.442
Text reading accuracy	0.26	*F*(6,445) = 26.33, *p* < 0.001	**< 0.001**	0.394	**< 0.001**	**0.020**	0.903	0.314
Word spelling	0.39	*F*(6,445) = 48.29, *p* < 0.001	**0.007**	**< 0.001**	**< 0.001**	0.306	0.176	0.160
Nonword spelling	0.05	*F*(6,445) = 4.20, *p* < 0.001	0.105	0.558	**0.022**	0.901	0.884	**0.001**
Text dictation	0.46	*F*(6,445) = 62.43, *p* < 0.001	**0.024**	**< 0.001**	**< 0.001**	0.255	0.954	0.160
Reading comprehension	0.19	*F*(6,445) = 17.95, *p* < 0.001	0.142	0.166	**< 0.001**	0.430	0.315	0.068

*Note: p* values < 0.05 are shown in bold.

Abbreviations: Diagn, diagnostic group; Lang, language background; SES, socioeconomic status.

#### 5.2.3 Cognitive and Language Measures

Nonverbal IQ explained 6% of the variance (*R* ^2^ = 0.06, *p* < 0.001). SES was significant (*p* = 0.001; *ηp* ^2^ = 0.02), as was SES × diagnostic group (*p* = 0.014; *ηp* ^2^ = 0.01). Simple effects showed that SES positively predicted Nonverbal IQ only in TD children (*β* = 0.33, *p* < 0.001), whereas no association was found in the SLD group (*p* = 0.522). Working memory explained 22% (*R* ^2^ = 0.22, *p* < 0.001) and was predicted by SES (*p* < 0.001; *ηp* ^2^ = 0.03) and diagnostic group (*p* < 0.001; *ηp* ^2^ = 0.11). Processing speed explained 5% (*R* ^2^ = 0.05, *p* = 0.002) and was predicted only by diagnostic group (*p* < 0.001; *ηp* ^2^ = 0.02). The model was not significant for nonword repetition (*R* ^2^ = 0.02, *p* = 0.315). Listening comprehension explained 10% of the variance (*R* ^2^ = 0.10, *p* < 0.001), with SES (*p* = 0.011; *ηp* ^2^ = 0.01) and diagnostic group (*p* < 0.001; *ηp* ^2^ = 0.03) as significant predictors, with no significant interactions (all *p*s > 0.1).

#### 5.2.4 Reading Measures

For word reading speed, the model explained 41% of the variance (*R* ^2^ = 0.41, *p* < 0.001), with a strong effect of diagnostic group (p < 0.001, *ηp* ^2^ = 0.36). Language background was not significant (*p* = 0.895). SES showed no main effect but interacted with diagnostic status (*p* = 0.033) and predicted faster reading speed only in TD children (*β* = 0.18, *p* = 0.002), whereas no association was found in the SLD group (*p* = 0.973). See Figure 3a. There was also a marginal interaction SES × language background (*p* = 0.059), where SES predicted word reading speed only in HB children (*p* = 0.024) but not in monolingual peers (*p* = 0.963).

**FIGURE 3:**
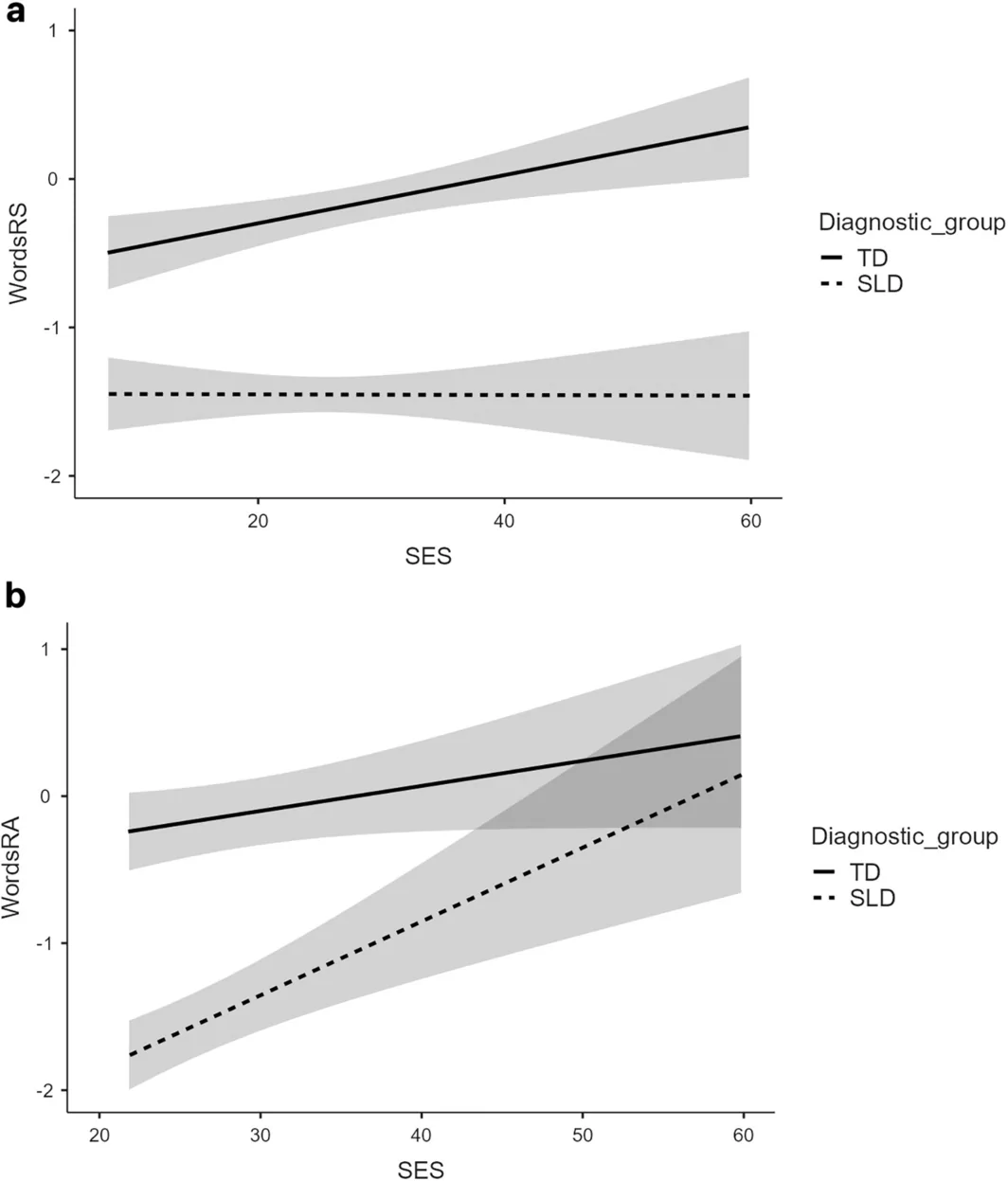
Interaction between SES and diagnostic group for word reading outcomes. Panel (a) shows the interaction between SES and diagnostic group (TD vs. SLD) in predicting word reading speed, indicating that higher SES is associated with faster reading only in typically developing children. Panel (b) shows the SES × diagnostic group interaction for word reading accuracy, where SES predicts accuracy in the SLD group but only marginally in the TD group. Error bands represent standard errors of the estimated marginal means.

For word reading accuracy, the model accounted for 24% of the variance (*R* ^2^ = 0.24, *p* < 0.001). SES (*p* < 0.001) and diagnostic group (*p* < 0.001) were significant predictors. Two interactions emerged: SES × diagnostic group (*p* = 0.020) and SES × language background (*p* = 0.046). The SES × diagnostic group interaction showed that SES predicted word reading accuracy in SLD children (*β* = 0.35, *p* < 0.001), whereas only a trend was found in the TD group (*β* = 0.12, *p* = 0.07). See Figure 3b. The SES × language background interaction (see Figure 4a) indicates that for bilinguals, higher SES was associated with more accurate word reading (*β* = 0.34, *p* < 0.001), whereas for monolinguals the association was weaker, although still significant (*β* = 0.13, *p* < 0.05). For nonword reading speed and accuracy, the models explained, respectively, 33% of the variance (*R* ^2^ = 0.33, *p* < 0.001) and 28% of the variance (*R* ^2^ = 0.28, *p* < 0.001). Diagnostic group was the only significant predictor (speed: *p* < 0.001, *ηp* ^2^ = 0.30; accuracy: *p* < 0.001, *ηp* ^2^ = 0.23). Neither SES nor language background contributed to performance (all ps > 0.05; see Table 4).

**FIGURE 4:**
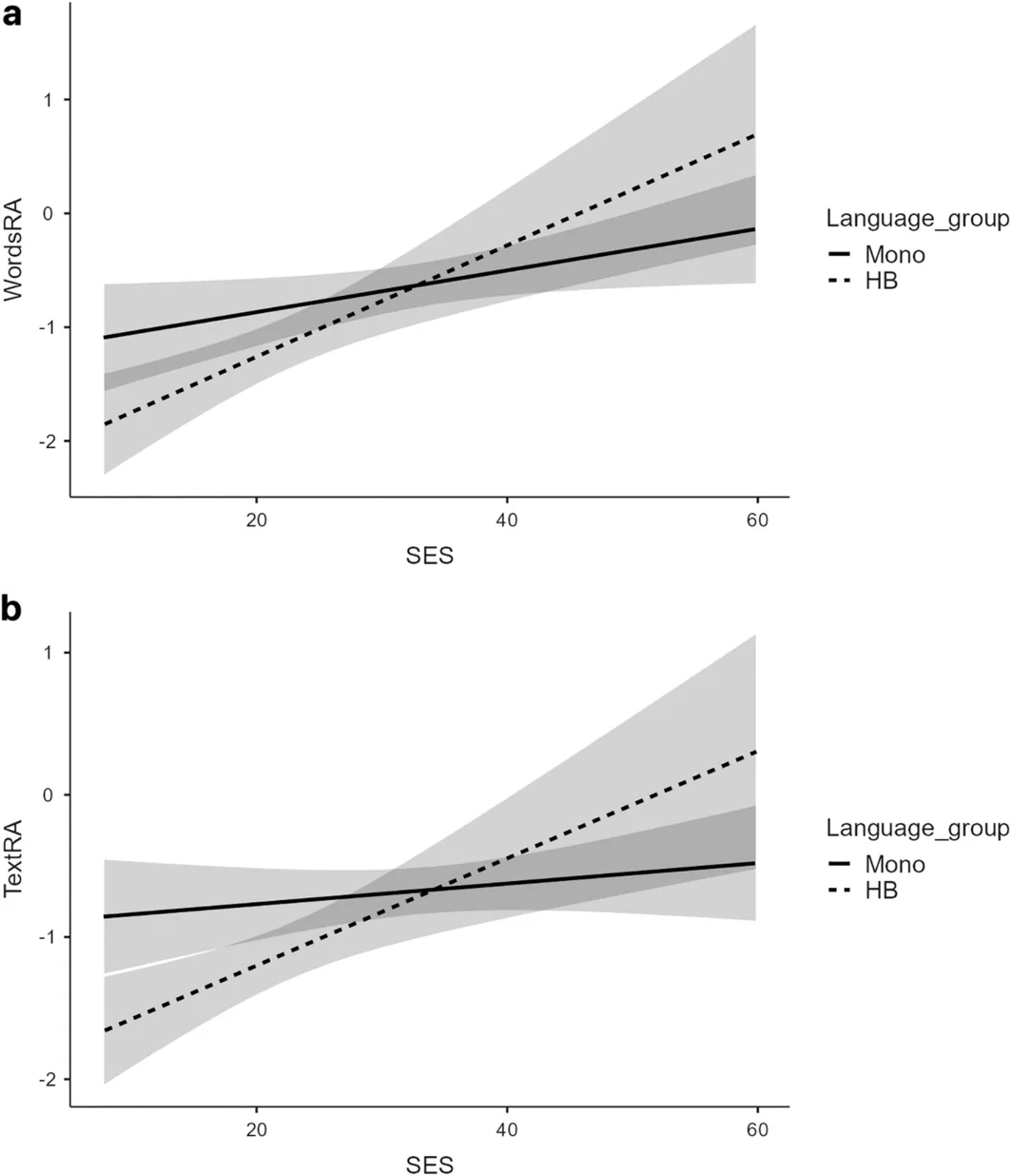
Interaction between SES and language background group for word and text reading accuracy outcomes. Panel (a) illustrates the interaction between SES and language background (heritage bilingual vs. monolingual) in predicting word reading accuracy, showing that higher SES is associated with greater accuracy primarily among bilingual children. Panel (b) displays the SES × language background interaction for text reading accuracy, revealing a similar pattern in which SES more strongly predicts accuracy in bilingual than in monolingual students. Error bands represent standard errors of the estimated marginal means.

For text reading speed the model explained 46% of the variance (*R* ^2^ = 0.46, *p* < 0.001). Diagnostic group was the only significant predictor (*p* < 0.001, *ηp* ^2^ = 0.42). Text reading accuracy explained 26% of the variance (*R* ^2^ = 0.26, *p* < 0.001), with significant effects of SES (*p* < 0.001; *ηp* ^2^ = 0.03) and diagnostic group (*p* < 0.001; *ηp* ^2^ = 0.17), and a SES × language background interaction (*p* = 0.02). SES was a significant predictor only for HBs (*β* = 0.30, *p* < 0.001), but not in monolinguals (*p* = 0.317) (see Figure 4b).

#### 5.2.5 Spelling Measures

For word spelling, the model explained 39% of the variance (*R* ^2^ = 0.39, *p* < 0.001). SES (*p* = 0.007; *ηp* ^2^ = 0.03), diagnostic group (*p* < 0.001, *ηp* ^2^ = 0.28), and language background (*p* < 0.001, *ηp* ^2^ = 0.03) were significant predictors. There were no significant interactions. For nonword spelling, the model explained 5% of the variance (*R* ^2^ = 0.05, *p* < 0.001). A main effect of diagnostic group (*p* = 0.022) was qualified by a significant diagnostic group × language background interaction (*p* = 0.001, *ηp* ^2^ = 0.02): HBs with SLD performed worse than monolingual children with SLD (*β* = 0.48, *p* = 0.002), whereas HB‐TD children tended to outperform the monolingual TD group (*p* = 0.057). For text dictation, the model explained 46% of the variance (*R* ^2^ = 0.46, *p* < 0.001). SES (*p* = 0.024; *ηp* ^2^ = 0.01), diagnostic group (*p* < 0.001; *ηp* ^2^ = 0.35), and language background (*p* < 0.001; *ηp* ^2^ = 0.04) were significant, with no significant interactions. Monolinguals outperformed bilinguals in both the TD (*p* = 0.021) and SLD (*p* < 0.001) groups, but with qualitatively larger differences in the SLD group.

#### 5.2.6 Reading Comprehension

Reading comprehension explained 19% of the variance (*R* ^2^ = 0.19, *p* < 0.001) predicted by diagnostic group (*p* < 0.001; *ηp* ^2^ = 0.14). The interaction diagnostic group × language background was not significant (*p* = 0.068); at the qualitative level, HBs with SLD scored lower than monolingual peers with SLD (*p* = 0.027), whereas TD groups scored the same (*p* = 0.786).

## 6 Discussion

The present study aimed to investigate the sociolinguistic profile of heritage bilingual (HB) children with and without specific learning disorders (SLD) in reading and spelling and to clarify how socioeconomic status (SES), language background, and diagnostic status jointly shape the literacy, language, and cognitive profiles of monolingual and HB children. These questions are particularly relevant in contemporary educational contexts where many children acquire literacy in a language other than their home language and where socioeconomic disparities intersect with multilingual development. In such situations, there is a risk of over‐ or under‐identification of SLD, and a widening of the lens is needed to understand the complex interplay between cognitive and environmental issues (Catts et al. 2024; Terry et al. 2023).

### 6.1 RQ1. Sociolinguistic Background of the Study Population

As expected from national data (ISTAT 2024), HBs had lower SES than monolinguals. Unexpectedly, lower SES was also found in the SLD group across both language groups. This pattern held across all SES components (parental education and occupation). Given the heritability of dyslexia (Pennington and Lefly 2001), this may reflect a cycle where parental learning difficulties limit educational and job opportunities, contributing to lower SES in line with the diathesis‐stress model (Scarr 1992). While some studies found no SES differences between SLD and non‐SLD families (Bonifacci et al. 2013; Dilnot et al. 2017), large cohort studies suggest SES does influence SLD diagnosis likelihood (Knight and Crick 2021). Although not a cohort study, this research, based on a sample drawn from Italian public health services, found that SLD diagnosis was more frequently associated with lower SES in both HB and monolingual children. While causal inferences cannot be made, this pattern warrants further investigation and highlights the potential role of socioeconomic factors in pathways to learning disorder identification.

In terms of sociolinguistic background, HBs had lower preschool attendance than monolinguals, a finding consistent with national data (MIM 2024). This may place HBs at a disadvantage at school entry, as they may have had fewer opportunities to develop basic skills and oral language through peer interaction. No differences were found between SLD and TD groups in the age of first words in Italian, but HBs spoke their first Italian words later than monolinguals. Within the HB group, SLD and TD children did not differ in age of L2 exposure, years since arrival (for children or parents), migration plans, or HL literacy exposure, indicating comparable family backgrounds. However, there were differences in language use. While both groups had similar HL and L2 exposure with parents, HB‐TD children showed more HL use with siblings and in media use. Their parents also reported higher Italian proficiency and more often rated their children as balanced bilinguals across comprehension, production, and preference, with a descriptively higher proportion of HL‐dominant ratings for production and comprehension. In contrast, HB‐SLD children were more Italian‐dominant in both use and reported proficiency (see Figure 2). These findings suggest that SLD may accelerate a shift towards the dominant language, particularly under conditions of lower SES. Although increased L2 use is often associated with stronger language and literacy skills (e.g., Carioti et al. 2024; Paradis 2023), this should not occur at the expense of the heritage language, as HL erosion can negatively affect socioemotional adjustment and family communication (De Houwer 2020). Consistent with previous findings showing a shift towards the majority language in families of children with neurodevelopmental disorders (Kay‐Raining Bird et al. 2016), often driven by social pressures (Borghetti et al. 2026), evidence specific to SLD remains limited and warrants further investigation.

Overall, our first research question shows that both HB children and those with SLD face greater social disadvantage than monolinguals and TD peers, and that HB children with SLD, despite sharing similar migration histories with HB‐TD children, display reduced maintenance of the HL.

### 6.2 RQ2. Multigroup Comparison and the Role of SES

The second research question examined the joint effects of SES, language background, and diagnostic status on cognitive and literacy outcomes. Preliminary models excluding SES suggested broader disadvantages for HBs, but these effects were largely reduced or eliminated once SES was included, indicating that SES accounted for most differences between bilingual and monolingual children.

Consistent with Hypothesis 2a, diagnostic status emerged as the strongest and most pervasive predictor across measures. Children with SLD showed clear difficulties in decoding, spelling, and reading comprehension, replicating established phonological and orthographic profiles, while nonverbal reasoning was preserved and weaknesses emerged in working memory and processing speed (e.g., Giofrè et al. 2017). Nonword repetition did not differ between diagnostic groups, in contrast to findings in developmental language disorders, particularly for HBs (Ortiz 2021). Overall, these results confirm the broad impact of diagnostic status and indicate that SLD‐related weaknesses are comparable in monolinguals and bilinguals.

As hypothesised (Hypothesis 2b), SES was positively associated with literacy and language outcomes, with domain‐specific effects. SES predicted listening comprehension, word and text reading accuracy, word spelling, and text dictation, but not reading speed or nonword reading and spelling, indicating a stronger role in tasks with higher lexical demands. In line with the dual‐route model (Coltheart et al. 2001), SES appears more closely related to the lexical route, which is sensitive to language exposure, than to the sublexical route based on phonological processing. SES also showed selective associations with cognitive measures, predicting verbal working memory across groups and nonverbal IQ only in typically developing children, but not processing speed or nonword repetition, which may rely more on basic processing efficiency and phonological short‐term memory. Finally, SES did not predict reading comprehension, contrary to previous findings (Ibáñez‐Alfonso et al. 2021; Bonifacci et al. 2022), possibly due to the stronger contribution of diagnostic status.

Regarding language background (Hypothesis 2c), once SES was controlled, bilingual and monolingual children showed comparable performance in decoding, reading and listening comprehension, nonverbal reasoning, verbal working memory, processing speed, and nonword repetition, consistent with previous findings on sublexical decoding accuracy and fluency in HBs (e.g., August and Shanahan 2006; Bonifacci and Tobia 2016). However, HBs showed weaker performance in word spelling and text dictation, in line with evidence of persistent difficulties in spelling and writing accuracy among HB learners, which may require longer consolidation in the school language (Affranti et al. 2024; Bonifacci et al. 2022). Spelling is particularly sensitive to the depth of vocabulary knowledge and the consolidation of word‐specific orthographic representations (Perfetti 2007), both of which depend on cumulative exposure to the school language. HB children, with relatively reduced Italian input compared to monolinguals, may therefore show spelling difficulties that partly reflect lower lexical quality rather than an underlying processing deficit per se. A more precise disentanglement of these two sources of variance would have required direct assessment of oral language proficiency in the heritage language, which was not available in the present study. Such measures could have shed light on the specific contribution of cross‐linguistic proficiency to spelling performance in HB children, a question that remains only partially addressed here and that future research should prioritise.

The observation that heritage bilingual children showed weaker performance in lexical spelling tasks (i.e., word spelling and text dictation), regardless of diagnostic status, suggests that these aspects of orthographic performance may represent a common feature of heritage bilingual learners acquiring literacy primarily in their second language. Notably, this pattern did not extend to nonword spelling, where typically developing HB children performed comparably to, or even tended to outperform, their monolingual peers. These findings indicate that lexical spelling performance should be interpreted with caution in this population, as lower accuracy may be associated with both bilingual language experience and neurodevelopmental factors. In line with the Italian National Guidelines issued by the Istituto Superiore di Sanità (2021), which recommend particular caution in the diagnostic assessment of spelling disorders in heritage bilingual children during primary school, our findings support the importance of complementing psychometric findings with careful consideration of the child's sociolinguistic background, language experience, and developmental trajectory when formulating a diagnostic judgement.

Finally, the inclusion of interaction terms (Hypothesis 2d) revealed patterns that would not emerge from simple group comparisons. For word and text reading accuracy, SES interacted with language background, predicting performance more strongly in HBs (and only in HBs for text reading accuracy), suggesting that reading accuracy in a second language is more sensitive to socioeconomic resources, possibly due to greater lexical demands and environmental influences. SES also interacted with diagnostic status in nonverbal reasoning, predicting nonverbal IQ only in typically developing children, but not in those with SLD, indicating that nonverbal reasoning in SLD may be more strongly constrained by neurodevelopmental factors, whereas it remains more sensitive to environmental variation in typical development.

For decoding skills, SES showed differential effects across reading dimensions: it was more strongly associated with word reading speed in the TD group, but with word reading accuracy in the SLD group. This pattern aligns with evidence from transparent orthographies showing that reading speed is a core marker of reading disorder and relatively resistant to environmental influences (Wimmer 1993), whereas accuracy may be more amenable to environmental support. Finally, the diagnostic group × language background interaction emerged only for nonword spelling and marginally for reading comprehension, indicating specific vulnerabilities in nonword spelling among HBs with SLD. This pattern mirrors findings on nonword repetition in developmental language disorders (Ortiz 2021) and suggests that nonword spelling may be a particularly sensitive indicator of SLD in HBs.

To summarise, diagnostic status emerged as the main source of variability across outcomes, whereas many bilingual–monolingual differences diminished once SES was taken into account. With SES controlled, HB and monolingual children showed comparable cognitive, linguistic, and decoding abilities. Differences related to language background appeared only in skills with higher lexical and orthographic demands, namely word spelling and text dictation, and, for HB‐SLD children only, nonword spelling. Overall, bilingualism was not associated with weaknesses in core decoding or cognitive skills but interacted with SES and SLD in ways that selectively affected more linguistically complex aspects of literacy.

### 6.3 Limitations

Several limitations should be acknowledged. First, assessments were conducted only in the second language (Italian), although best practices recommend testing HBs in both languages (Freeman and Schroeder 2022). This choice reflected the limited availability of standardised tools for heritage languages and the lack of formal literacy instruction in the HL for most children. The absence of direct HL proficiency data represents a constraint on the full interpretation of some outcomes. In particular, variability in HL oral proficiency could have contributed to individual differences in Italian literacy performance in ways not fully captured by the language history variables collected. This interpretive caveat is especially relevant for spelling outcomes, which are sensitive to the quality of lexical and phonological representations. As discussed above in relation to spelling outcomes, the contribution of language proficiency to literacy performance cannot be fully disentangled in the present study. Without direct HL assessment, it remains unclear to what extent the observed spelling difficulties in HB children reflect a genuine literacy disorder versus the cumulative effect of reduced exposure to Italian as the school language. Two considerations, however, partially mitigate this concern. First, all SLD diagnoses were established prior to recruitment through multidisciplinary clinical assessments conducted within the Italian National Health Service, following procedures that explicitly require ruling out insufficient language exposure as a primary cause of academic difficulties. Second, and importantly, HB children with SLD showed markedly lower lexical spelling scores than their HB‐TD peers despite the absence of significant differences in language history variables between the two groups, including age of exposure, length of residence, and language use patterns. This pattern suggests that the observed difficulties are unlikely to be explained solely by differences in language exposure, and points instead to genuine processing weaknesses. Future studies should therefore include oral proficiency measures in both languages alongside literacy assessments, in order to further separate language‐experience effects from neurodevelopmental ones and to establish more accurate bilingual benchmarks for SLD identification. Secondly, more fine‐grained measures of language proficiency in the societal language, such as vocabulary depth, morphosyntactic knowledge, and listening comprehension, would have allowed a more precise examination of the relationship between L2 oral language skills and literacy outcomes, particularly spelling. Finally, the sample was linguistically and culturally heterogeneous; future studies should consider linguistic distance and analyse reading and spelling error patterns to better capture language‐specific effects.

## 7 Conclusions and Implications

This study advances previous research by examining HB and monolingual children with and without SLD within a single framework, demonstrating that language background, neurodevelopmental status, and SES jointly shape literacy and cognitive outcomes. By providing a detailed characterisation of sociolinguistic background, including SES, language exposure, and heritage‐language use, it addresses a gap in the literature and highlights a potential risk of heritage‐language erosion in HBs with SLD, with implications for policy and practice. The inclusion of SES and interaction terms reveals underexplored patterns, such as the stronger role of SES in L2 reading accuracy and in tasks with higher lexical demands, underscoring the importance of incorporating SES into literacy research (Singh and Rajendra 2024). Moreover, the study offers one of the few direct examinations of the literacy phenotype of HBs with SLD.

Overall, these findings emphasise the need to interpret HB children's literacy performance within a broader clinical and sociolinguistic context. Socioeconomic disadvantage may contribute to diagnostic bias in SLD, particularly among HBs, and should not limit access to support; in such cases, educational interventions targeting literacy stimulation may be more beneficial than an exclusive focus on diagnostic labelling. Furthermore, the finding that spelling difficulties extend to typically developing HB children reinforces the need for caution in applying monolingual normative thresholds in bilingual contexts. Consistent with Italian national guidelines (ISS 2021), formal SLD diagnosis in HB children should not rely solely on psychometric cut‐offs but should integrate the child's full sociolinguistic trajectory, consider deferring classification until sufficient literacy consolidation in the school language has occurred, and prioritise clinical judgement over threshold‐based criteria alone. Finally, the greater heritage‐language use observed in typically developing children further suggests that balanced bilingualism and heritage‐language maintenance may act as protective factors, supporting recommendations to encourage family‐language use in children with SLD. Overall, assessment in bilingual contexts should move beyond monolingual norms (Lai et al. 2025), combining detailed linguistic histories with the development of bilingual benchmarks to ensure appropriate tools and expectations that reflect the complexity of bilingual development.

## Funding

The authors have nothing to report.

## Ethics Statement

The study was approved by the Ethics Committee approval for each site of the multicenter study (632–2021‐OSS‐AUSLBO; 977–2021‐OSS‐AUSLBO; 900/2021/Oss/AUSLF; 734–2021‐OSS‐AUSLIM; 840/2021/OSS/AUSLMO; 848/2021/OSS/AUSLPR; 971/2021/OSS/AUSLPC; 8540/2021/I.5/228/Cerom; 869/2021/OSS/AUSLRE).

## Consent

Written informed consent for participation and data processing was obtained from both parents of all participating children.

## Conflicts of Interest

The authors declare no conflicts of interest.

## Supporting information


**Data S1:** Statistical output of preliminary analyses (ANOVAs) with language background (HBs vs. monolinguals) and SLD (SLD vs. TD) as the main factors and the main study measures as the dependent variables.

## Data Availability

The data that support the findings of this study are available on request from the corresponding author. The data are not publicly available due to privacy or ethical restrictions.
