# Supplementary material for: Socioeconomic Status and Fine‐Grained Literacy Outcomes in Heritage Bilingual and Monolingual Children With and Without Specific Learning Disorders
Source: Dyslexia. 2026 Oct 9;32(4):e70051. doi: 10.1002/dys.70051 (PMC13652706; doi:10.1002/dys.70051)
Supplement: Supplementary file 1 [file DYS-32-e70051-s001.docx]

**Supplementary material.** Statistical output of preliminary analyses (ANOVAs) with language background (HBs vs. monolinguals) and SLD (SLD vs. TD) as the main factors and the main study measures as the dependent variables

|  | SLD | LB |
| --- | --- | --- |
| Non verbal IQ. | *F*_(1, 453)_= 1.28, *p*= .26; n_p_^2^ = .003; | *F*_(1, 453)_= 1.58, *p*= .21; n_p_^2^ = .004; |
| Working Memory index: | ***F*_(1, 453)_= 86.79, *p*<.001; n_p_^2^ = .16;** | ***F*_(1, 453)_= 14.45, *p*<.001; n_p_^2^ = .03;** |
| Processing Speed | **F(1, 453)= 15.51, p<.001; np2 = .03;** | *F* _(1, 453)_ < 1 |
| Listening Comprehension | ***F*_(1, 453)_= 24.64, p<.001; n_p_^2^ = .05,** | ***F*_(1, 453)_= 7.53, p<.01; n_p_^2^ = .02;** |
| Nonword repetition | ***F*_(1, 453)_= 5.41, p<.05; n_p_^2^ = .01.** | *F* _(1, 453)_ < 1 |
| Word reading accuracy | ***F*_(1, 453)_= 145.09, *p <*.001; _np_^2 =^ .24** | ***F*_(1, 453_) = 9.65, *p <*.01; _np_^2 =^ .02** |
| Word reading speed | ***F*_(1, 453_) = 386.19, *p <*.001; _np_^2 =^ .46** | *F* _(1, 453)_ < 1 |
| Nonword reading accuracy | ***F*_(1, 453)_= 207.58, *p <*.001; _np_^2 =^ .32** | *F* _(1, 453)_ < 1 |
| Nonword reading speed | ***F*_(1, 453)_= 255.98, *p <*.001; _np_^2 =^ .36** | ***F*_(1, 453)_= 6.74, *p =*.01; _np_^2 =^ .02^** |
| Text reading accuracy | ***F*_(1, 453_) = 153.36, *p <*.001; _np_^2 =^ .25** | ***F*_(1, 453_) = 7.26, *p <*.01; _np_^2 =^ .16** |
| Text reading speed | ***F*_(1, 453_) = 532.72, *p <*.001; _np_^2 =^ .54** | *F* _(1, 453)_ < 1 |
| Word spelling | ***F*_(1, 453_) = 347.9, *p <*.001; _np_^2 =^ .44** | ***F*_(1, 453_) = 27.94, *p <*.001; _np_^2 =^ .06** |
| Nonword Spelling | ***F*_(1, 453_) = 23.249, *p <*.001; _np_^2 =^ .05** | *F* _(1, 453)_ < 1 |
| Text dictation | ***F*_(1, 453_) = 437.47, *p <*.001; _np_^2 =^ .49** | ***F*_(1, 453_) = 46.61, *p <*.001; _np_^2 =^ .09** |
| Reading comprehension | ***F*_(1, 453_) = 125.96, *p < .001*; _np_^2 =^ .22** | *F*_(1, 453_) = 2.66, *p =* .1; _np_^2 =^ .006 |

Notes: in bold statistically significant results; ^ HB > Monolinguals
